# Towards an integrated systems-based modelling framework for drug transport and its effect on tumour cells

**DOI:** 10.1186/1754-1611-8-3

**Published:** 2014-01-13

**Authors:** Cong Liu, Krishnan, Xiao Yun Xu

**Affiliations:** 1 Department of Chemical Engineering, Imperial College London, South Kensington Campus, London SW7 2AZ, UK; 2 Centre for Process Systems Engineering and Institute for Systems and Synthetic Biology, Imperial College London, South Kensington, London SW7 2AZ, UK

**Keywords:** Drug transport, Drug effect, Intracellular signalling, *in silico* experimental platform, Systems-based modelling framework

## Abstract

**Background:**

A systematic understanding of chemotherapeutic influence on solid tumours is highly challenging and complex as it encompasses the interplay of phenomena occurring at multiple scales. It is desirable to have a multiscale systems framework capable of disentangling the individual roles of multiple contributing factors, such as transport and extracellular factors, and purely intracellular factors, as well as the interactions among these factors. Based on a recently developed systems-based modelling framework, we have developed a coupled system in order to further elucidate the role of drug transport, and its interplay with cellular signalling by incorporating intra- and extra-vascular drug transport in tumour, dynamic descriptions of intracellular signalling and tumour cell density dynamics.

**Results:**

Different aspects of the interaction between transport and cell signalling and the effects of transport parameters have been investigated *in silico*. Limited drug penetration is found to be a major constraint in inducing drug effect; many aspects of the interaction of transport with cell signalling are independent of the details of cell signalling. A sensitivity analysis indicates that the effect of drug diffusivity depends on the balance between interstitial drug transport and the specific requirement for triggering apoptosis (governed by highly nonlinear signalling networks), suggesting that the effect of drug diffusivity in such cases must be considered in conjunction with descriptions of cellular dynamics.

**Conclusions:**

The modelling framework developed in this study provides qualitative and mechanistic insights into the effect of drug on tumour cells. It provides an *in silico* experimental platform to investigate the interplay between extracellular factors (e.g. transport) and intracellular factors. Such a platform is essential to understanding the individual and combined effects of transport and cellular factors in solid tumour.

## Introduction

The efficacy of chemotherapy is strongly dependent on the transport of anticancer drugs to tumour cells and their responses to the administrated drug [1]; both can be compromised significantly by complexities associated with cancer, which is a disease of collective dysregulations across multiple scales.

To exert therapeutic effects, anticancer drugs must reach tumour cells with a sufficiently high concentration [2]. Limited penetration is one of the major causes of failure of chemotherapy treatment of solid tumours. The first obstacle to most blood-borne chemotherapeutic agents is posed by the abnormal and chaotic tumour vasculature, which limits tumour blood flow and consequently the supply of drugs and nutrients [3]. After crossing the capillary wall, anticancer drugs must penetrate through the tumour interstitium, where drug distribution is determined by the effectiveness of drug transport by diffusion and convection as well as drug consumption [2]. Elevated interstitial fluid pressure in solid tumours hinders convective transport, rendering diffusion the dominant mechanism for interstitial drug transport [4]. Drug diffusivity depends strongly on the physicochemical properties of the specific drug, such as molecular weight, shape, charge and solubility. Drug consumption involves drug binding, sequestration and metabolism, which can be altered by microenvironmental conditions, such as extracellular matrix composition and structure, cell packing density and the presence of tumour acidity [3,5,6].

For most anticancer drugs, it is necessary for drug molecules to transport across cell membranes to reach the target molecules and interact with them, as a consequence, triggering cellular signal transduction. Cellular signalling is one of the important characteristics of every living cell in that it governs the basic cellular activities by perceiving and correctly responding to external/internal stimuli. Anticancer drugs as stress stimuli can regulate/trigger cell signalling to kill cells or affect their cellular responses (cell apoptosis, cell proliferation, differentiation and migration), which might be directly associated with fatal consequences of cancer [7-9]. The way by which cellular signalling functions is extremely complex as cellular pathways are not isolated from each other but are interconnected through a complex network [10]. In particular, highly non-linear input-output relationships are usually displayed in cell signalling networks, with a number of emergent properties, such as adaptive responses and robust switching by positive feedback [11,12]. It is worth emphasizing that the dynamic interactions and signal transmission in these chemical networks control cellular decision making and cellular responses such as movement, apoptosis etc.

Considerable effort has been devoted to developing mathematical models to predict drug concentration and drug effect on solid tumours (for a review, see [13]). Compartmental models have been widely adopted for prediction of temporal profiles of drug concentration in designed compartments, particularly drug concentration in blood in pharmacokinetic studies [14-16]. To obtain spatio-temporal drug distributions in a given tumour geometry, it is necessary to explicitly account for drug transport (diffusion or/and convection) [17-19]. After drug concentrations are obtained, suitable pharmacodynamic models can be used to predict the effect of drug as a function of drug concentration and/or as a function of time following drug administration in a phenomenological and empirical manner, with elaboration of observed data thus neglecting detailed underlying mechanisms [20-22]. On the other hand, deterministic models can be used to describe the tumour response by assuming a drug concentration-dependent tumour growth characteristic or tumour death kinetics [23-25]. Unfortunately, mathematical models addressing the above mentioned areas have been developed separately; furthermore, they often bypass a key component that is the dynamic process of cellular signal transduction. While all these models are capable of providing certain levels of insights, none of them offers a transparent and integrated description of drug transport and drug effect accounting for the associated cellular signalling.

In this study, an integrated systems-based mathematical modelling framework is employed and extended, which captures the information flow from drug delivery to the outcome, thus including biological transport processes of drugs and cellular response and accounting for dynamics of the relevant signal transduction. This allows us to begin to probe and elucidate different aspects of the roles and the interaction of transport and intracellular signalling dynamics. In a spatially distributed system, intracellular signalling is triggered in response to heterogeneous drug stimuli delivered through transport pathways. It must be emphasized here that drug stimuli are dynamic (being delivered through complex vascular networks), while drug transport can be affected by many tissue level features and intracellular dynamics is highly nonlinear. Finally the dynamic coupling of these factors is not necessarily unidirectional. For instance the tissue scale properties and features could be a potential factor in affecting drug transport; as an example, it is found that apoptosis-inducing pretreatment enhances drug delivery [26,27]. Furthermore, with considerable progress in unravelling the intracellular and intercellular signal transduction in systems biology, mathematical modelling approaches can begin to go beyond empirical and transport centric models to integrate dynamic descriptions of transport and cellular signalling for a systematic examination of their interactions.

This paper examines some basic aspects of the interaction of transport and cellular dynamics. As a first step, the model is formulated on a simplified geometry of tumour vasculature, in which explicit coupling of blood flow between vascular and interstitial space is incorporated, along with drug transport. The effects of anticancer drugs are addressed by integrating the above with dynamics of intracellular apoptosis signalling. The integrated model is used to evaluate treatment strategies and to analyse other factors that may influence the response of tumour cells, in order to provide insights into the complex interplay between the different processes involved.

## Methods

For mathematical modelling of drug transport, a commonly adopted approach is to avoid an explicit representation of the tumour vasculature which, instead, is treated as a distributed source term in the governing equations [19,28-31]. In doing so, descriptions of transport processes are incomplete without accounting for vascular transport and the spatial relationship between blood vessels and tumour interstitium. However, incorporating realistic tumour vasculature geometry is highly challenging, given the fact that the tumour vasculature is abnormal, irregular and heterogeneous. Further complexities in evaluating drug effects are added when dynamic intracellular signalling processes are incorporated, which are triggered in response to spatio-temporal drug stimuli and exhibit highly non-linear dynamics. To obtain clear-cut and transparent insights into transport mechanisms, cellular signalling and their interaction, we employ the modelling framework as an *in silico* experimental platform which describes a well-defined tumour-drug system with minimal essential elements, definite information flow and a controlled source of variability and heterogeneity. The *in silico* experimental platform depicts an idealized tumour with no heterogeneity, in a simplified geometry. This setup is designed as an initial effort to contain the minimal components necessary for understanding the effects of drugs on tumours and elucidating the effects of transport and cellular factors in a transparent manner without consideration of other factors.

### Computational geometry

**Figure 1:**
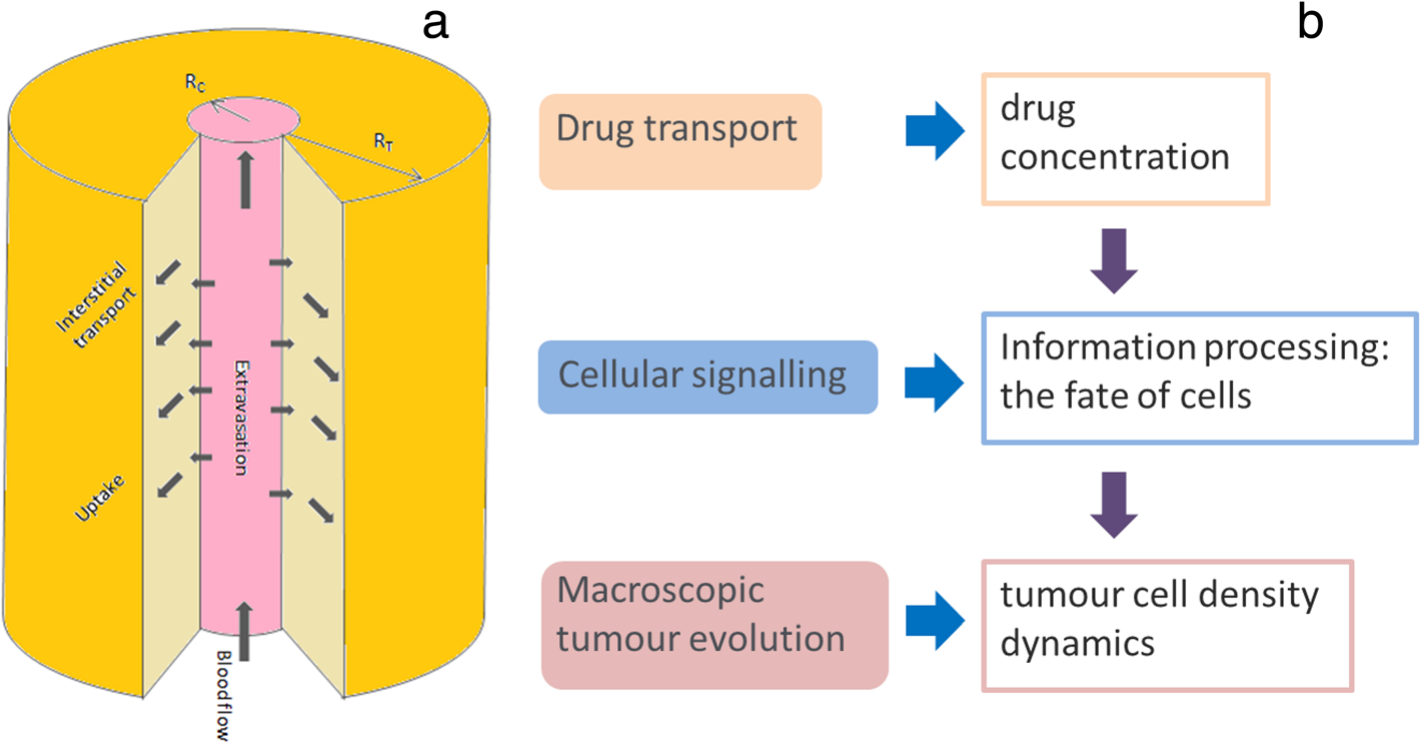
**Schematic overview of information flow involved in the modelling framework. (a)** Schematic illustration of the simplest tumour vascular geometry and **(b)** diagrammatical representation of the levels of description incorporated in the model.

The model consists of a single blood vessel surrounded by the tumour interstitium, which is a simplified representation also employed in previous studies [32,33]. Although the geometrical configuration is similar to that of a tumour cord model [17,18,34], they differ in size in that the present model mimics the entire transport domain in the tumour tissue. While the equivalent vessel geometry and the corresponding parameters adopted in the model may be an over-simplification, it is practical and tractable to start with this simple geometry for the purpose of understanding the essential interplay between drug transport and drug effect. Figure 1 depicts the computational geometry and the flow of information in the model.

### Mathematical models

**Figure 2:**
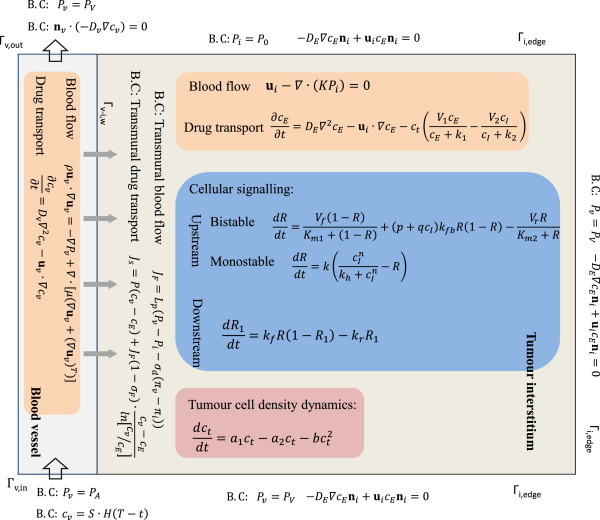
**Overview of mathematical descriptions involved in each domain with corresponding boundary conditions.** Details of biological transport (blood flow as well as drug transport), coarse-grained cellular apoptosis signal transduction and tumour cell density dynamics refer to following subsections.

The current modelling framework includes basic descriptions of blood flow, drug transport, intracellular apoptosis signalling and tumour cell density dynamics. The main assumptions are as follows: (1) blood is an incompressible, Newtonian fluid and the blood vessel is straight and rigid; (2) the tumour interstitium is homogeneous, with a uniform distribution of nutrients and pH; (3) tumour cells are stationary, leading to the assumption that the tumour interstitium has a fixed outer boundary; (4) all tumour cells are distributed uniformly, identical (i.e. ignoring cellular variability, stochasticity, and the effects of cell-to-cell interactions and cell-cycle) and alive initially. These assumptions are made with the understanding that the key ingredients are reasonably represented in the initial model and that individual assumptions may be relaxed in subsequent studies. Detailed descriptions of each of the elements are given in the following sections. A brief overview of the mathematical equations for different regions in the computational domain is presented in Figure 2, with symbols and values of parameters defined in Tables 1 and 2.

**Table 1:** Parameters and values used in the mathematical models

**Parameter**	**Symbol**	**Value**	**Reference**
**Tumour blood flow**			
Tumour capillary radius	R_C_	100 (μm)	[33]
Tumour interstitium radius	R_T_	1200 (μm)	Estimated
Tumour length	L	1 (cm)	[33]
Blood density	*ρ*	1000 (kg/m^3^)	[32]
Blood viscosity	*μ*	0.004 (Pa∙s)	[35]
Vascular hydraulic conductivity	*L*_*p*_	0.36-2.8 × 10^-6^ (cm/mmHg∙s)	[36]
Tissue hydraulic conductivity	*K*	0.4-2.0 × 10^-7^ (cm^2^/mmHg∙s)	[36]
Vascular inlet pressure	*P*_*A*_	15 (mmHg)	[37]
Vascular outlet pressure	*P*_*V*_	0	[36]
Ambient pressure	*P*_*0*_	0	[36]
**Drug transport (Doxorubicin)**	*D*_*E*_	1.5778 × 10^-6^ (cm^2^/s)	[17]
DOX diffusion coefficient			
Rate of transmembrane transport	*V*_*1*_, *V*_*2*_	0.28 (ng/(10^5^cells)/min)	[14]
	(V_1_ = V_2_)		
Diffusive permeability for DOX	*P*_*E*_	2.778 × 10^-4^ (cm/s)	[17]
Michaelis constant for transmembrane transport	*k*_*1*_	0.219 (μg/ml)	[14]
Michaelis constant for transmembrane transport	*k*_*2*_	1.37 (ng/(10^5^cells))	[14]
**Tumour cell density**			
Initial tumour cell density	*c*_*t,0*_	10^6^ (cells/mm^3^)	[17]
Tumour cell growth rate	*a*_*1*_	0.5 (day^-1^)	Estimated
Saturation constant in logistic equation	*b*	0.02592 (mm^3^/(10^5^cells)/day)	Estimated
Tumour cell natural decay rate	*a*_*2*_	0.24 (day^-1^)	Estimated
**Intracellular signalling:**			
**Bistable switch**			
Michaelis Menten constants	*V*_*f*_	27 (hr^-1^)	[38]
Michaelis Menten constants	*V*_*r*_	0.459 (hr^-1^)	[38]
Michaelis Menten constants	*K*_*m1*_	100	[38]
Michaelis Menten constants	*K*_*m2*_	0.01	[38]
Kinetic parameter mediating feedback strength	*k*_*fb*_	2.927 (hr^-1^)	[38]
Basal level in the bistable switch	*p*	0.7	Estimated
Parameter mediating input regulation in the bistable switch	*q*	0.3(ng/(10^5^cells))^-1^	Estimated
**Monostable switch**			
Kinetic parameter reflecting the time scale of the response	*k*	0.432 (hr^-1^)	Estimated
Associated constant	*k*_*h*_	1 (ng/(10^5^cells))	Estimated
Hill coefficient	*n*	10	Estimated
R_1_ protein activation rate	*k*_*f*_	3.6 (hr^-1^)	Estimated
R_1_ protein degradation rate	*k*_*r*_	0.144 (hr^-1^)	Estimated
R_1_ Threshold for apoptosis switch	*R*_*1*_*,*_*th*_	0.9	Estimated

**Table 2:** Variables and their definitions in the mathematical models

**Symbol**	**Definition**	**Symbol**	**Definition**
**u**_*v*_	Blood velocity vector in vascular domain	*c*_*v*_	DOX concentration in vascular domain
**u**_*i*_	Blood velocity vector in interstitial domain	*c*_*E*_	Extracellular DOX concentration
*P*_*v*_	Vascular blood pressure	*c*_*I*_	Intracellular DOX concentration
*P*_*i*_	Interstitial fluid pressure	*R*	Hypothetical downstream intermediate protein in apoptosis signalling cascade
*J*_*F*_	Transmural fluid velocity, determined by Starling’s law	*R*_1_	Hypothetical protein responsible for triggering apoptosis
*J*_*s*_	Transmural drug flux, determined by Kedem-Katchalsky equation	*c*_*t*_	Tumour cell density

#### Tumour blood flow

The model accounts for the coupling between vascular, transmural and interstitial fluid flow since tumour blood vessels are highly permeable. Blood flow is assumed to be steady, which is acceptable here as it is capable of serving as a fundamental platform to investigate the dynamic behaviour of drug transport and intracellular events without imposing further complexities related to pulsatile blood flow.

Flow within the blood vessel is governed by the Navier-Stokes equations:

(1a) $$\rho u_{\nu }\cdot \nabla u_{\nu }=‒\nabla P_{\nu }+\nabla \cdot \left[\mu \left(\nabla u_{\nu }+{\left(\nabla u_{\nu }\right)}^{T}\right)\right]$$

(1b) $$\nabla u_{v}=0$$

Where *ρ* is blood density, *μ* is blood viscosity, **u** is blood velocity vector with subscript *v* denoting vascular space, and *P*_*v*_ is vascular blood pressure.

Eqn. 1a and Eqn. 1b are solved subject to the following boundary conditions:

(2a) $$P_{v}=P_{A}\;\mathrm{on}\;\Gamma_{v,\mathrm{in}}$$

(2b) $$P_{v}=P_{V}\;\mathrm{on}\;\Gamma_{v,\mathrm{out}}$$

(2c) $$n_{v}\cdot u_{v}=J_{F}\;\mathrm{on}\;\Gamma_{v-i,w}$$

Fluid motion in the interstitial space is described by Darcy’s law:

(3a) $$u_{i}-\nabla \cdot \left(KP_{i}\right)=0$$

(3b) $$\nabla u_{i}=0$$

where *K* represents interstitial hydraulic conductivity, and **u**_i_ and *P*_*i*_ are blood velocity vector and fluid pressure in the interstitium.

The boundary conditions for Eqn. 3a and Eqn. 3b are:

(4a) $$‒n_{i}\cdot u_{i}=J_{F}\;\mathrm{on}\;\Gamma_{v-i,w}$$

(4b) $$P_{i}=P_{0}\mathrm{on}\;\Gamma_{i,\mathrm{edge}}$$

The boundary conditions (BC) described by Eqn. 2a and Eqn. 2b specify a constant arterial and venous pressure at the inlet and outlet of the vessel, respectively; BC (2c) assumes a transmural velocity in the normal direction (outward) of the blood vessel. BC (4b) assigns the ambient pressure at all boundaries of the interstitium (except the vessel wall); BC (4a) prescribes a transmural velocity normal (inward) to the vessel wall. The transmural velocity, *J*_*F*_ can be calculated using Starling’s law:

(5) $$J_{F}=L_{p}\left(P_{v}-P_{i}-\sigma_{d}\left(\pi_{v}-\pi_{i}\right)\right)\;\mathrm{on}\;\Gamma_{v‒i,w}$$

Where *L*_*p*_ is vascular hydraulic conductivity, *σ*_*d*_ is osmotic reflection coefficient, and *π*_*v*_ and *π*_*i*_ are osmotic pressure in the vascular and insterstitial space, respectively. As the contribution of osmotic pressure difference is negligible in solid tumours, Starling’s law is reduced to

(6) $$J_{F}=L_{p}\left(P_{v}-P_{i}\right)\;\mathrm{on}\;\Gamma_{v‒i,w}$$

#### Drug transport

Three variables are considered in the drug transport processes: the intravascular drug concentration (*c*_*v*_), the interstitial extracellular free drug concentration (*c*_*E*_), and intracellular drug concentration (*c*_*I*_). Within the blood vessel, the drug is transported mainly by convection with negligible reactions (e.g. metabolism, binding/unbinding, uptake). Once extravasated into the tumour interstitium, drug particles penetrate through the intersititum via diffusion and convection and at the same time they may be taken up by tumour cells. The drug is treated as a blood-borne solute and its transport is governed by a diffusion-convection-reaction equation.

**Solute dynamics in the blood vessel (Ω**_**v**_**)** is governed by:

(7) $$\frac{\partial c_{v}}{\partial t}=D_{v}\nabla^{2}c_{v}-u_{v}\cdot \nabla c_{v}$$

Where *c*_*v*_ refers to drug concentration in the vascular space, and *D*_*v*_ is drug diffusivity in the vascular space. The boundary conditions are:

(8a) $$c_{v}=S\cdot H\left(T-t\right),\;t>0\;\mathrm{on}\;\Gamma_{v,\mathrm{in}}$$

(8b) $$n_{v}\cdot \left(-D_{v}\nabla c_{v}\right)=0\;\mathrm{on}\;\Gamma_{v,\mathrm{out}}$$

(8c) $$-D_{v}\nabla c_{v}n_{v}+u_{v}c_{v}n_{v}=-J_{s}\;\mathrm{on}\;\Gamma_{v‒i,w}$$

BC (8a) prescribes a pulse injection at the vessel inlet with a constant intensity *S* and infusion time *T*, in which Heaviside term *H*() indicates infusion occurs during the period of t = 0 to t = *T*. BC (8b) defines a convective flux at the outlet; BC (8c) sets an outward solute flux across the leaky wall boundary.

**Solute dynamics in the interstitium (Ω**_**i**_**)** is governed by the extracellular and intracellular drug transport.

Extracellular drug concentration:

(9a) $$\frac{\partial c_{E}}{\partial t}=D_{E}\nabla^{2}c_{E}-u_{i}\cdot \nabla c_{E}-c_{t}\left(\frac{V_{1}c_{E}}{c_{E}+k_{1}}-\frac{V_{2}c_{I}}{c_{I}+k_{2}}\right)$$

Where *c*_*E*_ and *c*_*I*_ refer to the extracellular and intracellular drug concentration, respectively, *D*_*E*_ is diffusion coefficient of drug in the interstitium, while *c*_*t*_ is tumour cell density. *V*_*1*_, *V*_*2*_, *k*_*E*_ and *k*_*I*_ are constants that describe transport across the cell membrane, in which *V*_*1*_ and *V*_*2*_ are the maximum rates of transmembrane transport, while *k*_*1*_ and *k*_*2*_ are the Michaelis-Menten constants for transmembrane transport. Eqn. 9a describes diffusion, convection of extracellular drugs and their uptake/pumping out by tumour cells with the last terms expressed by Michaelis-Menten kinetics. The boundary conditions are:

(10a) $$-D_{E}\nabla c_{E}n_{i}+u_{i}c_{E}n_{i}=0\;\mathrm{on}\;\Gamma_{i,\mathrm{edge}}$$

(10b) $$-D\nabla c_{E}n_{i}+u_{i}c_{E}n_{i}=J_{S}\;\mathrm{on}\;\Gamma_{v-i,w}$$

BC (10a) describes a no flux condition at the other boundaries of the interstitium. In the current study, neither the surrounding host tissue nor functional lymphatics in the host tissue are explicitly described. However, it is possible to incorporate the effect of lymphatic drainage in the current set up by imposing a mixed boundary condition at the outer surface. Simulations with both BCs gave essentially identical results for the stimuli used here (results not shown). The effect of the host tissue will be explicitly examined in a future study. BC (10b) assigns an inward solute flux into the interstitium, which can be determined by the Kedem-Katchalsky equation:

(11) Js=Pcv-cE+JF1-σF△clm⋯△clm=cv-cElncvcEonΓv-i,w

Where *P* is drug diffusive permeability across the vessel wall, *σ*_*f*_ is osmotic reflection coefficient, Δ*c*_*lm*_ is the log-mean concentration across the vessel wall, and *J*_*F*_ is the fluid flux across the vessel wall, which is determined by Starling’s law.

### Intracellular drug concentration

The intracellular drug concentration depends on the corresponding extracellular drug concentration according to transmembrane transport. This is given by

(12) $$\frac{\partial c_{I}}{\partial t}=\frac{V_{1}c_{E}}{c_{E}+k_{1}}-\frac{V_{2}c_{I}}{c_{I}+k_{2}}$$

It is noted that drug binding to plasma proteins (mainly albumin) is neglected in the current study. In many cases, the binding/unbinding process is described simply by first-order kinetics; therefore its effect is relatively simple in that it reduces the amount of free extracellular drug available to tumour cells. Incorporation of drug binding would not alter the system output qualitatively, but would dramatically increase computational burden. However, it is essential to take this into account when quantitative modelling is required or sophisticated binding mechanisms are involved.

#### Intracellular apoptosis signalling

The modelling of intracellular signalling processes, a substantial core of systems biology, plays a very important role in the entire modelling framework as decisions on cell fate are processed and determined by molecular signalling networks. It is thus very important to have a dynamic representation of this in the modelling framework. Given the complexity of signalling which contains many missing biological details and unknown parameters, it is essential to choose an appropriate level of description in the model to start with, so that the most important known aspects of the signalling and cellular decision making are included. Thus, the strategy adopted here is to start with coarse grained descriptions of the cellular signalling dynamics, which are capable of representing correctly the nature of the information flow, and ensuring that the qualitatively important features of detailed models are accounted for. We believe that this is more appropriate than starting with detailed models containing many unknown factors and other details whose correctness may be difficult to establish. At the same time, this provides a platform that allows more detailed mechanistic models to be incorporated in the future.

When modelling the intracellular processes in response to chemotherapy, the main process of interest is apoptosis (programmed cell death) following the administration of anticancer agents. Based on systems biology investigations and existing models [39-45], we recognize that two key features must be reflected by any model regardless of its complexity. Firstly, some threshold effect must be present; secondly, the “switch” to apoptosis must be realized in an irreversible way.

The apoptosis models adopted here are based on the two types of switches commonly observed in cellular signalling: bistable and monostable apoptosis switches. As bistable switches can exhibit irreversibility intrinsically, they have been used in modelling irreversible cell fate decision-making in apoptosis [40,43,45]. Generally, positive feedback (biologically existing in the intracellular caspase network) and cooperativity (in apoptosome formation) are regarded as sources of bistability. However, as far as apoptosis is concerned, it is not obvious whether representing the irreversible fate (cell death) as a steady state is necessary or even reasonable (at the cell fate decision level). It is possible that an irreversible decision is made when critical cellular events are triggered, from which there is no turning back. Thus the irreversibility could result from a simple irreversible reaction, which is kept under tight control and triggered only under very special circumstances [39,41,42]. Noting this, and the fact that both mechanisms have been discussed in the context of apoptosis, the two models employed in this study are: (1) a bistable switch with self-contained threshold behaviour and irreversibility; (2) a sequential interconnection of a monostable switch and a downstream irreversible reaction effect [34].

### Bistable switch

(13a) $$\frac{\mathrm{dR}}{\mathrm{dt}}=\frac{V_{f}\left(1-R\right)}{K_{m1}+\left(1-R\right)}+\left(p+qc_{I}\right)k_{\mathrm{fb}}R\left(1-R\right)-\frac{V_{r}R}{K_{m2}+R}$$

Where *K*_*m1*_ and *K*_*m2*_ are the Michaelis-Menten parameters. *k*_*fb*_ is a kinetic parameter which parametrizes the feedback strength. The constants *p* and *q* serve to set the basal level and dynamic range of the module.

### Irreversible monostable switch

(13b) $$\frac{\mathrm{dR}}{\mathrm{dt}}=k\left(\frac{c_{I}^{n}}{k_{h}+c_{I}^{n}}-R\right)$$

Where *n* denotes the Hill coefficient, *k*_*h*_ an associated constant in the Hill term, and *k* is a parameter representing the time scale of the response.

In both models, *R* acts as a typical downstream intermediate element along the signal transduction while *R*_*1*_ represents the output responsible for directly triggering apoptosis.

(14) $$\frac{dR_{1}}{\mathrm{dt}}=k_{f}R\left(1-R_{1}\right)-k_{r}R_{1}$$

Once a threshold of *R*_*1*_ is crossed, cell death is triggered. Normalised concentrations of molecules *R* and *R*_*1*_ are adopted. Two additional points are worth emphasizing here. Firstly, the actual choice of monostable/bistable model has a very minor effect, as analysis with other model variants has led to very similar results. Secondly, detailed characterization and comparison of monostable and bistable models demonstrate their similarities and differences, which provide a basis for their use in contexts such as this [46].

#### Tumour cell density dynamics

The equation governing the tumour cell density is described by a continuous logistic equation [47]:

(15) $$\frac{dc_{t}}{\mathrm{dt}}=a_{1}c_{t}-a_{2}c_{t}-bc_{t}^{2}$$

Where *a*_*1*_ is tumour growth rate, *a*_*2*_ is tumour natural decay rate and *b* is a saturation constant in the logistic tumour growth equation.

Eqn. 15 naturally describes the growth and death of cells with saturating growth effect leading to a finite steady state (models with some variations have very similar net results: see [46]). These equations provide an explicit representation of the key features of interest. Although the population balance formalism provides a more comprehensive description of birth and death of cells, it is computationally highly demanding and can be difficult to handle if additional cellular complexities are included.

When tumour cells are perturbed by anticancer drugs, the intracellular apoptosis signalling is initiated, resulting in cell death at the population level. Since cell density is described in a continuous, rather than discrete form, triggering of the intracellular threshold is represented by a sharp fall in growth rate at the population level. Clearly, the dynamics of the logistic model imply that if the growth rate becomes sufficiently low, the zero steady state (*c*_*t*_ = 0) would be the only biologically relevant state, indicating that all cells will eventually die. It should be noted that computational implementation of this threshold effect is achieved in a reversible way in the bistable model, but in a unidirectional (irreversible) way in the monostable model.

#### Initial conditions

Except for tumour cell density, all other variables are set to be zero initially. A uniform tumour cell density is assumed prior to drug injection.

### Numerical methods

#### Computational procedures

All numerical simulations presented in this paper are implemented in the finite element based software Comsol Multiphysics. The simulation of blood flow is decoupled from that of drug transport and tumour cell density by assuming the velocity field is independent of the drug concentration field and tumour cell density distribution. Steady-state simulation of blood flow is carried out first. Upon obtaining the pressure and velocity fields, drug transport is resolved by solving the diffusion-convection-reaction equation. The boundary conditions at the vessel wall are implemented in accordance with the physical settings in Comsol Multiphysics. With regard to fluid flow, transmural velocity (*J*_*F*_) is positive in both the vessel and interstitial domain as it points away from the surface in the vessel domain, and at the same time, it represents the inflow to the interstitial domain. *J*_*F*_ is set as a variable in accordance with Starling’s law, which enables the coupling of the vascular fluid pressure (*P*_*v*_) to the interstitial fluid pressure (*P*_*i*_). For drug transport, an inflow flux is set by default, which means a negative transmural flux (*J*_*S*_) in the vessel domain, and a positive flux in the interstitial domain. Intracellular signal transduction is triggered by the local intracellular drug concentration, the response of which is manifested through tumour cell density owing to decreased tumour growth rate or increased tumour death rate. The tumour cell density, in turn, affects drug transport.

The equations are discretised and solved on a pre-generated computational mesh. Mesh sensitivity study is carried out first to provide mesh independent solutions. The final mesh consists of 75,000 and 325,000 mapped meshes for drug transport and tumour cell density, respectively.

#### Model parameters

Values of all parameters as well as variables used in the integrated model are defined in Tables 1 and 2, respectively. These are extracted from a variety of sources as they span multiple scales of description and are not available in a single tumour-drug system. Values for blood flow related parameters are mainly extracted from similar mathematical models found in the literature. Doxorubicin (DOX) is one of the anticancer drugs commonly used in clinics and a large amount of experimental data is available on its physical and pharmacokinetics properties; therefore it is chosen as a representative anticancer drug for parameterization purpose. Values for tumour growth parameters are chosen to reflect both the range of steady state, as well as the appropriate time scale. In the intracellular dynamics, parameters are not generally available and their values are determined based on appropriate reflection of the time scales involved in apoptosis signalling. Further, the downstream threshold (*R*_*1,th*_) is specially chosen to ensure complete *R*_*1*_ activation while maintaining the upstream signal for a sufficient time period. This in turn directly reflects the cellular behaviour to exposure of drugs for a sufficient time. Thus our intracellular descriptions are parameterized so that they are capable of triggering apoptosis decisions for comparable signals as are seen experimentally. It should be emphasized that most of the essential conclusions drawn from this study are not strongly dependent on the particular numerical choice of parameters.

## Results

In this section, numerical results of blood flow are presented first, followed by results of drug transport and distribution. The effect of drug examined in terms of tumour cell density distribution by considering both bistable and irreversible monostable intracellular apoptosis models under various pulse drug injections. In addition, a sensitivity analysis is performed on parameters involved in drug transport. Results for drug concentration and tumour cell density are presented in the dimensionless form, which are normalised by their corresponding reference values: 0.001 μg/mm^3^ for vascular and extracellular drug concentrations, 1 ng/10^5^cells for intracellular drug concentration and 10^6^ cells/mm^3^ for tumour cell density.

### Blood flow

**Figure 3:**
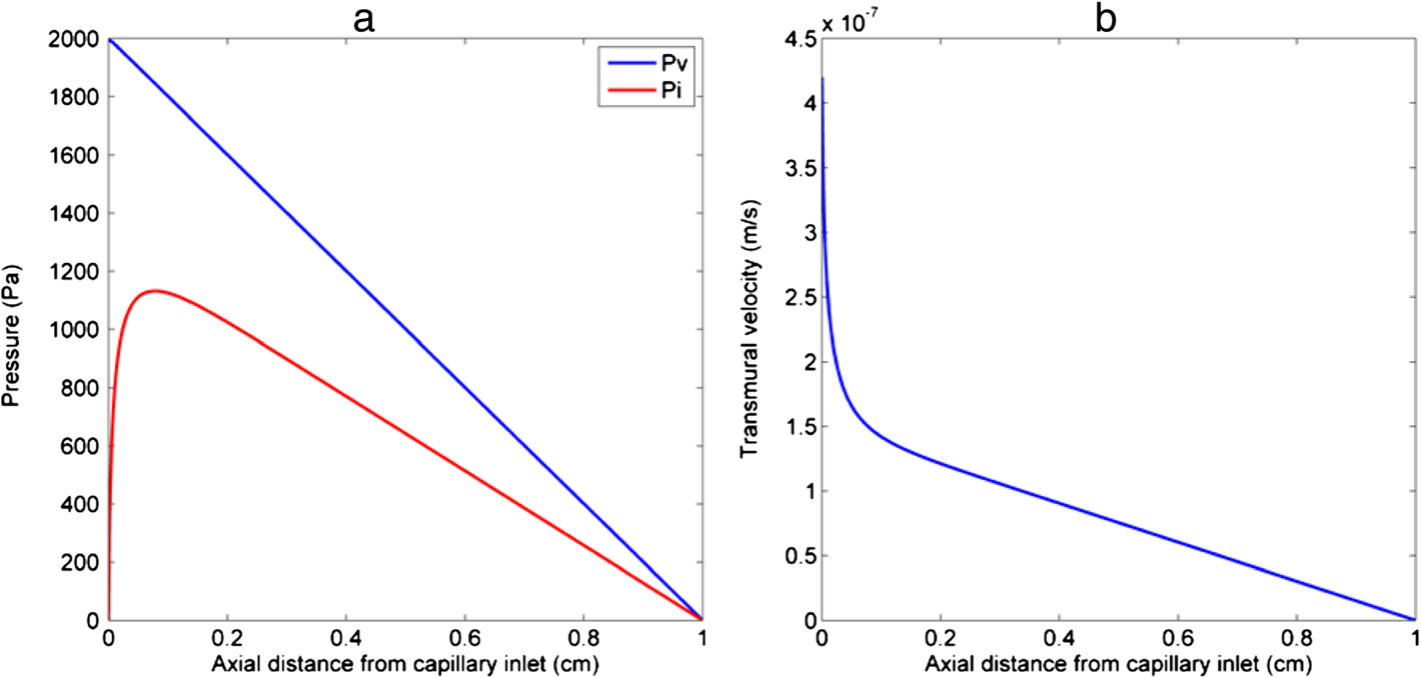
**Axial distribution of (a) vascular (*P***_***v***_**) and interstitial fluid pressure (*P***_***i***_**) on the capillary wall, (b) transmural velocity.**

Axial pressure profiles on the lumen and tissue side of the blood vessel wall together with the transmural velocity are presented in Figure 3. It is seen that vascular pressure falls linearly along the flow direction while interstitial fluid pressure experiences a sharp rise near the vessel inlet, followed by a gradual reduction. This is due to the entrance effect caused by the discontinuity of pressure conditions imposed at the vessel inlet and the adjacent lower boundary of the interstitium. The simulation results demonstrate that interstitial fluid pressure is strongly coupled to vascular fluid pressure due to elevated hydraulic conductivity normally found in tumour tissues. Since the pressure difference across the capillary wall determines the filtration velocity of blood, the latter (as shown in Figure 3(b)) experiences a sharp fall near the vessel inlet before declining gradually along the flow direction as the transmural pressure difference (*Pv*-*Pi*) diminishes.

### Drug transport and distribution

#### Drug transport

Drugs are transported in tumour tissues by diffusion and convection. The relative importance of diffusion and convection is measured by Peclet number (*Pe*) which is defined as:

$$\mathrm{Pe}=\frac{\mathrm{Lu}}{D}$$

where L is the characteristic length, *u* is a representative velocity, and *D* is the diffusion coefficient for a given anticancer drug. Since *D* is 1.578 × 10^-10^ m^2^/s for doxorubicin (Table 1), the corresponding *Pe* in the blood vessel is on the order of 10^6^, suggesting that intravascular drug transport is dominated by convection.

Drugs extravasate into the tumour interstitium by diffusion and convection, determined by diffusive permeability *P* and blood filtration velocity *J*_*F*_. The filtration velocity shown in Figure 3(b) is of the order of 10^-7^ m/s, which is an order of magnitude lower than diffusive permeability (2.78 × 10^-6^ m/s) across the vessel wall for doxorubicin, suggesting that the total transmural flux is dominated by the diffusive flux in this case.

**Figure 4:**
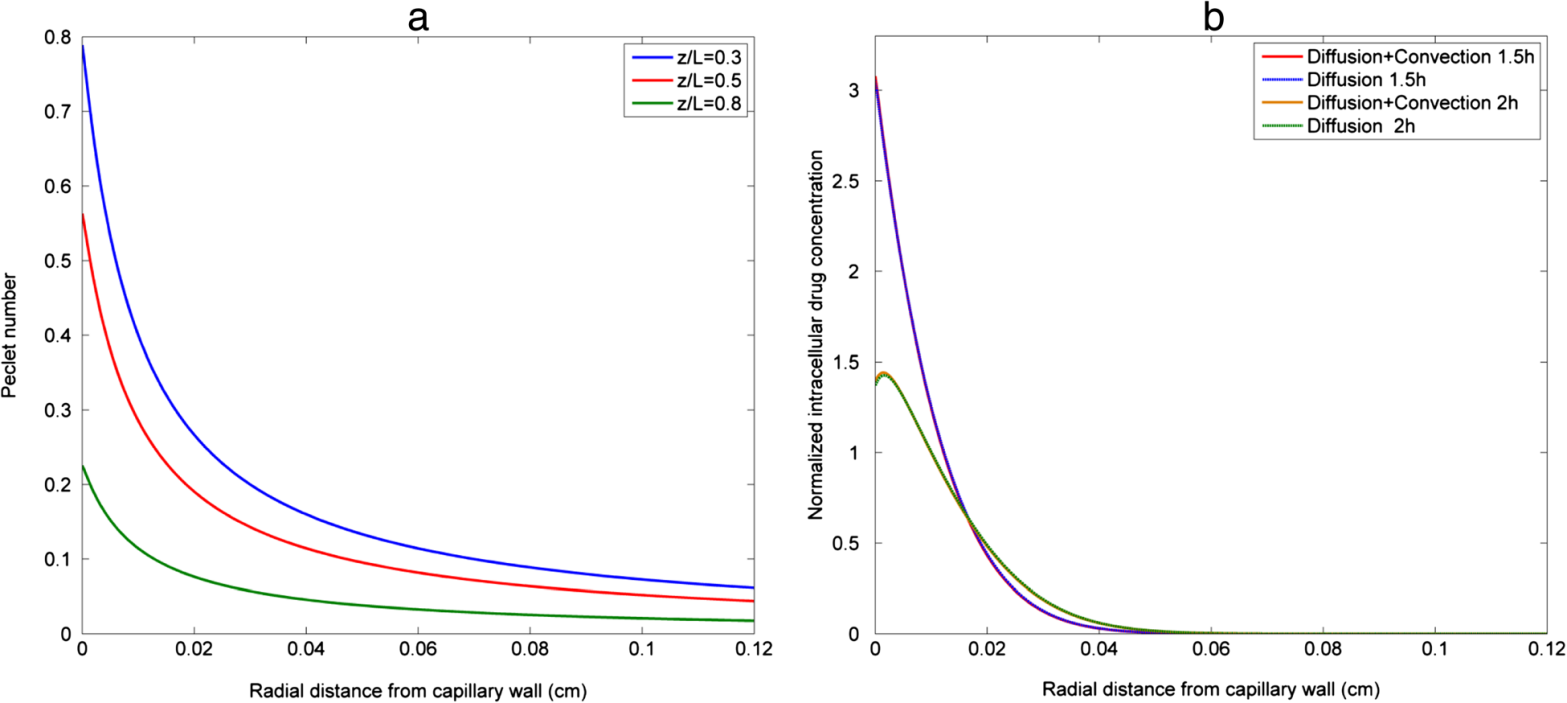
**Role of diffusion and convection in drug transport. (a)** cross-sectional profiles of *Pe* number at various axial positions in tumour interstitium, **(b)** quantitative comparison of normalized intracellular drug concentration at axial location z/L = 0.5 with and without convection under baseline pulse injection at t = 1.5 h (end of injection) and at t = 2 h (0.5 h after injection). Pulse characteristics of baseline pulse: pulse intensity *S* = 1, infusion time *T* = 1.5 h.

With regard to interstitial drug transport, variations of *Pe* (based on local radial velocity) along the radial direction at three different axial locations are displayed in Figure 4(a). It shows that *Pe* is generally of the order of 10^-1^ and decreases along both the radial and axial directions, which suggests that diffusion is becoming more dominant than convection and the role of convection may be limited and confined to a region close to the wall. Furthermore, with the use of a simple tumour vascular geometry, the effect of convection can be quantified by comparing cross-sectional profiles of intracellular drug concentration. As shown in Figure 4(b), the difference is almost negligible, which confirms that diffusion plays a dominant role in interstitial drug transport.

#### Drug distribution

**Figure 5:**
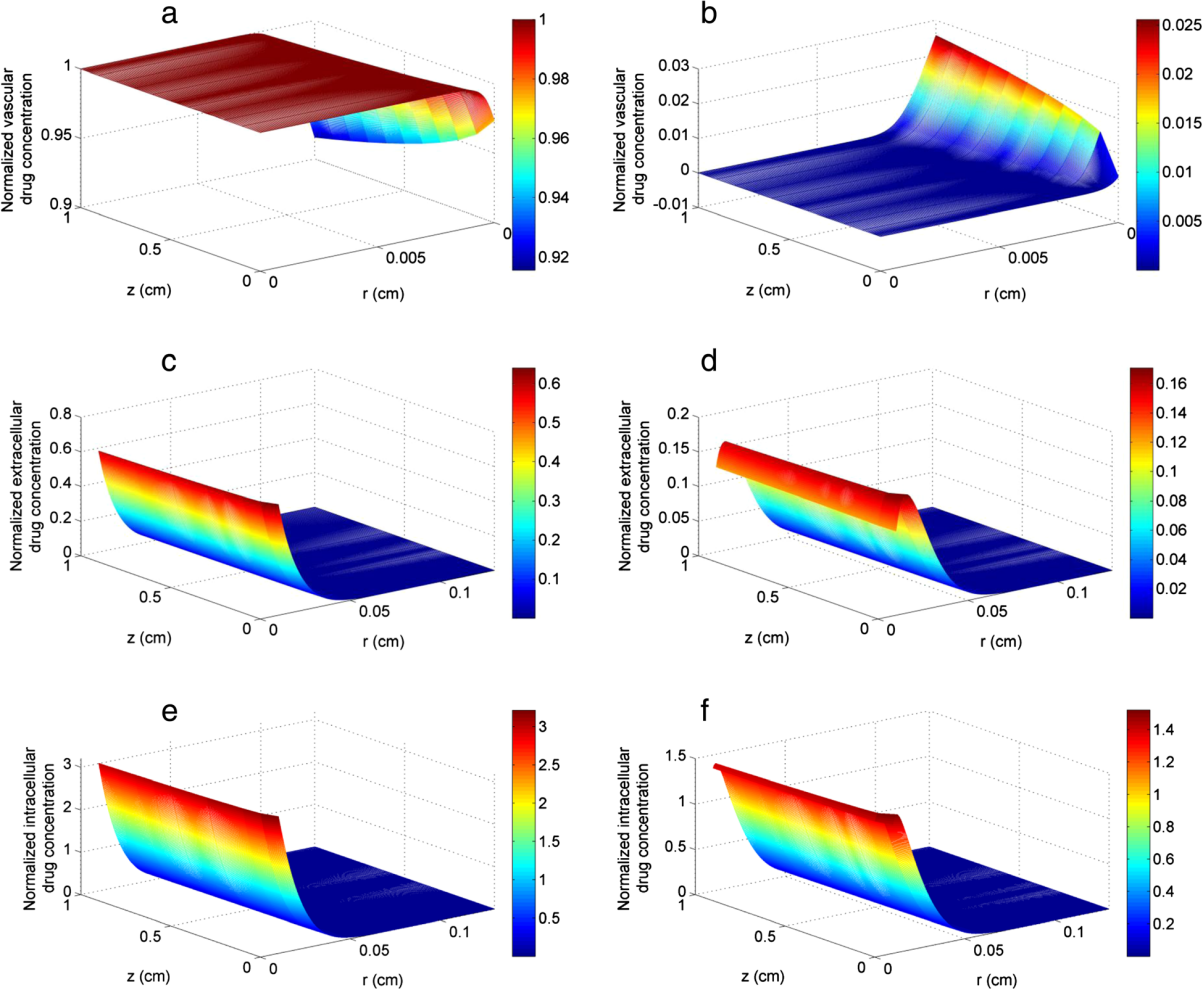
**Snapshots of spatial profiles of drug concentration under baseline pulse injection for bistable switch.** Upper panel: vascular drug concentration at **(a)** t = 1.5 h, **(b)** t = 2 h. Middle panel: extracellular drug concentration at **(c)** t = 1.5 h, **(d)** t = 2 h. Bottom panel: intracellular drug concentration at **(e)** t = 1.5 h, **(f)** t = 2 h.

The anticancer drug is assumed to be directly injected into the blood stream at the inlet of the blood vessel in the form of a pulse, which is an appropriate type of signal as it represents the time-dependent nature without introducing further complexity to the analysis of dynamic interactions between tumours and drugs. For a systemic administration, a more realistic drug input expressed as an exponentially decaying function of time [48,49] or based on injection details could be readily incorporated in the future. Snapshots of spatial profiles of drug concentration are displayed in Figure 5, where t = 1.5 h corresponds to the end of pulse injection and t = 2 h is 0.5 h after drug injection. In Figure 5(a) drug concentration is uniform in the core region, while a concentration boundary layer is seen near the wall with drug concentration on the inner vessel wall decreasing along the direction of blood flow (z direction). Figure 5(c) shows that a steep extracellular drug concentration gradient is established close to the vessel wall while little drug reaches beyond 5*R*_*C*_ (*R*_*C*_ is the equivalent vessel radius). As intracellular drug concentration is dependent on the local extracellular drug concentration, it follows the same trend as shown in Figure 5(e) but with a larger value due to the kinetics of transmembrane transport.

Displayed in Figure 5(b,d,f) are snapshots of vascular, extracellular and intracellular drug concentrations at t = 2 h, half an hour after drug injection. In response to the sudden termination of drug input, reversal of concentration gradient is observed in the near wall region inside the blood vessel and in the interstitium. In this context, the interstitium acts as a reservoir, from which drugs are transported back to the blood vessel and eventually leave the blood vessel by convection. The reverse transport of drugs is confined to a thin layer close to the vessel wall, while drugs outside this layer are transported outward in the radial direction by diffusion and convection. Therefore, the extracellular drug concentration profile experiences a rise and reaches a peak before falling off; the same is observed for the intracellular drug concentration profile.

### Drug effect--Tumour cell density distribution

#### Baseline case

**Figure 6:**
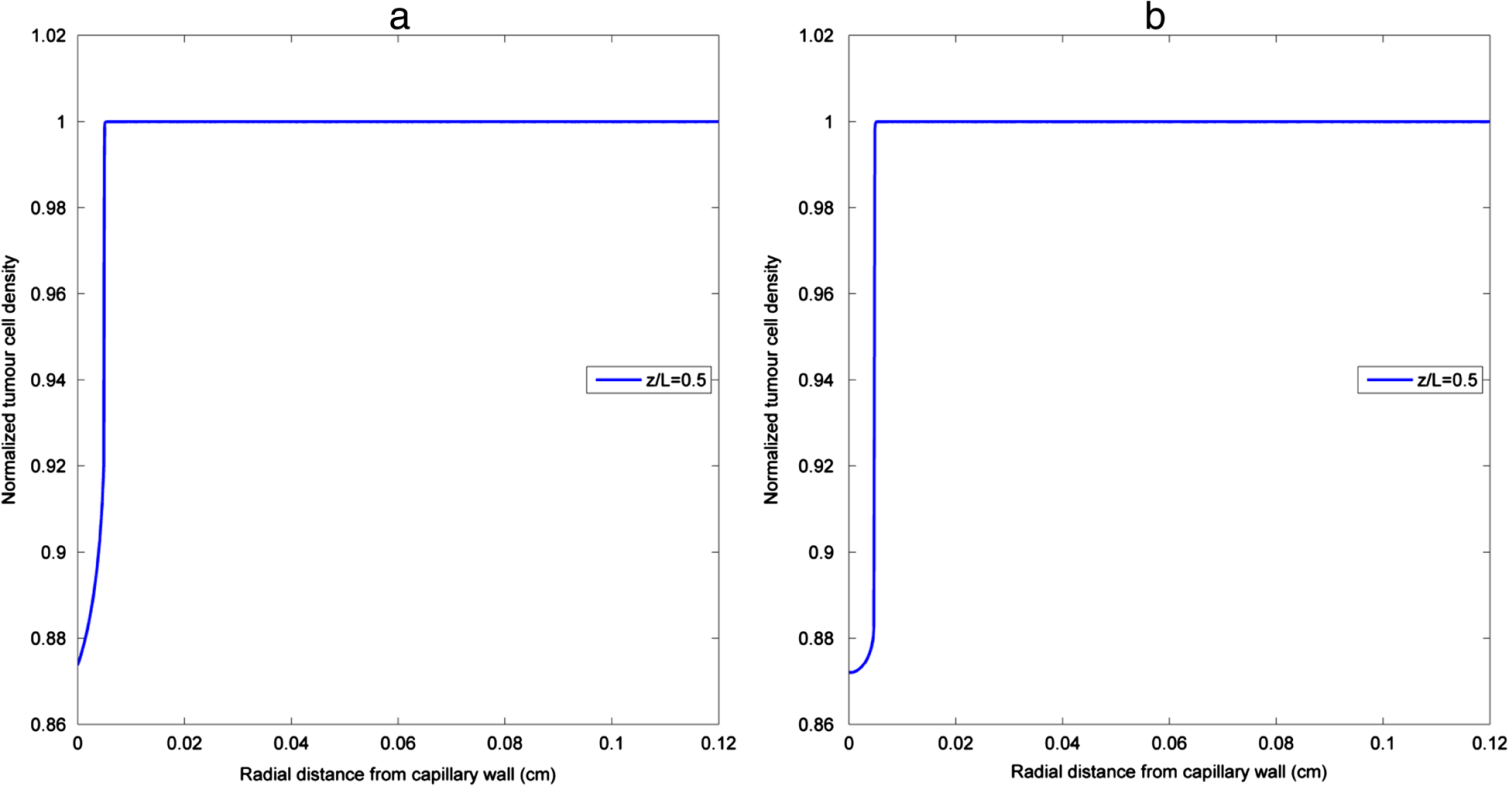
**Tumour cell density under baseline pulse injection.** Cross-sectional profiles at z/L = 0.5 of tumour cell density at 10 h display similar trends for **(a)** bistable switch, **(b)** irreversible monostable switch.

As cell apoptosis is triggered by effective intracellular drug concentration (above the threshold) which shows little variation in the axial direction, tumour cell density at any cross section away from the inlet and outlet effects (e.g. z/L = 0.5) would be sufficiently representative and can be used to compare results obtained with different simulation parameters. Displayed in Figure 6(a) and (b) is the distribution of tumour cell density under the same pulse injection for bistable and irreversible monostable switches respectively. As effective drug penetration is confined to the close vicinity of the vessel wall, cell death only occurs in a small region, leaving most of the tumour interstitial space unaffected. Similar results are predicted by both bistable and monostable apoptosis switches. Although intracellular drug concentration is above its threshold transiently for a pulse injection, irreversibility of the intracellular apoptosis module ensures that tumour cell density continues to decrease in the cell killing region, implying that a single pulse can only kill cells in a confined region and the width of tumour cell death region at a specific time point (e.g. the instant when triggering of apoptosis is completed) is capable of reflecting the drug effect. Therefore, in the following results, relative tumour cell death regions are presented for bistable and irreversible monostable apoptosis switches to illustrate the effects of pulse properties, different pulse fractionations and other influencing parameters.

#### Effects of pulse infusion time and intensity

**Figure 7:**
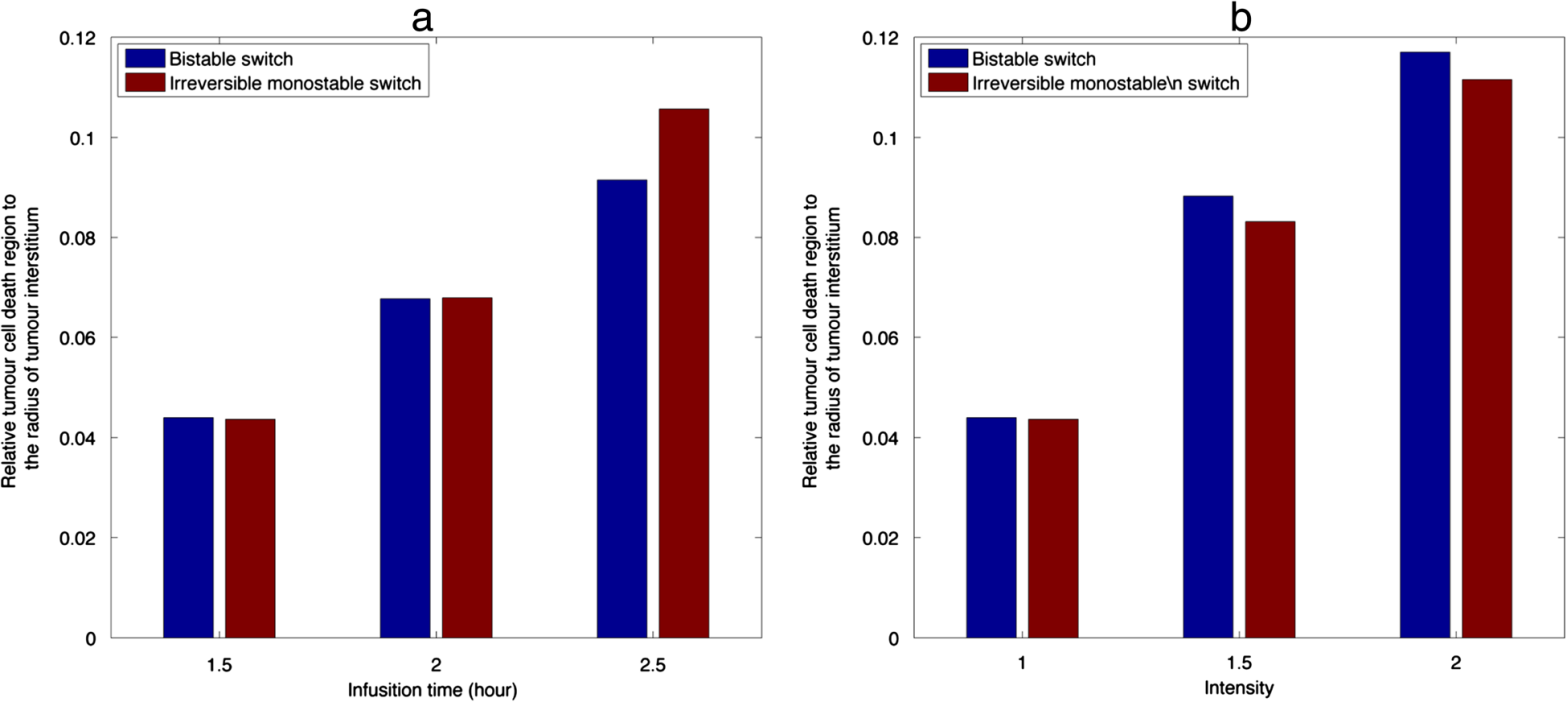
**Effect of pulse characteristics on the relative tumour cell death region at 10 h. (a)** Effect of infusion time under baseline pulse intensity. **(b)** effect of pulse intensity under baseline infusion time.

Figure 7(a) shows the effect of infusion time on the width of tumour cell death region for bistable and irreversible monostable switches. For both cases, an increase in infusion time causes a moderate extension of the cell killing region, as a result of improved extracellular drug transport. A similar effect is found with increased pulse intensity, as shown in Figure 7(b).

The predicted tumour cell density shown here has a weaker non-linear response to increases in infusion time or pulse intensity compared to our previous study [34]. Although the *in silico* experiments in both studies use the same basic modelling elements, they differ in the geometrical scale of interest and the level of integration between different elements; the former may significantly affect the role of drug transport and intracellular signalling in cell killing. In our previous study [34], the time scale for transport (diffusion) in a microscopic tumour cord geometry is of the order of seconds, and the results suggest that transport over such a small time scale plays a less important role, which results in a more obvious nonlinear response. However, in the present *in silico* simulations, a relatively large tumour interstitium is adopted to mimic drug penetration through the entire tumour tissue. Within this context, the time scales for drug transport (dominated by diffusion) and intracellular signalling are comparable; therefore, the transport limitation poses a major constraint in inducing the effect of anticancer drugs since reactions involved in cellular signalling are triggered by intracellular drug concentration. It is concluded that limitations in transport can act as a buffer to reduce the sensitivity of cell-killing region to changes in the characteristics of stimuli. This is further demonstrated through the sensitivity analysis on the size of tumour domain (presented in sensitivity analysis section).

#### Effects of pulse fractionations

**Figure 8:**
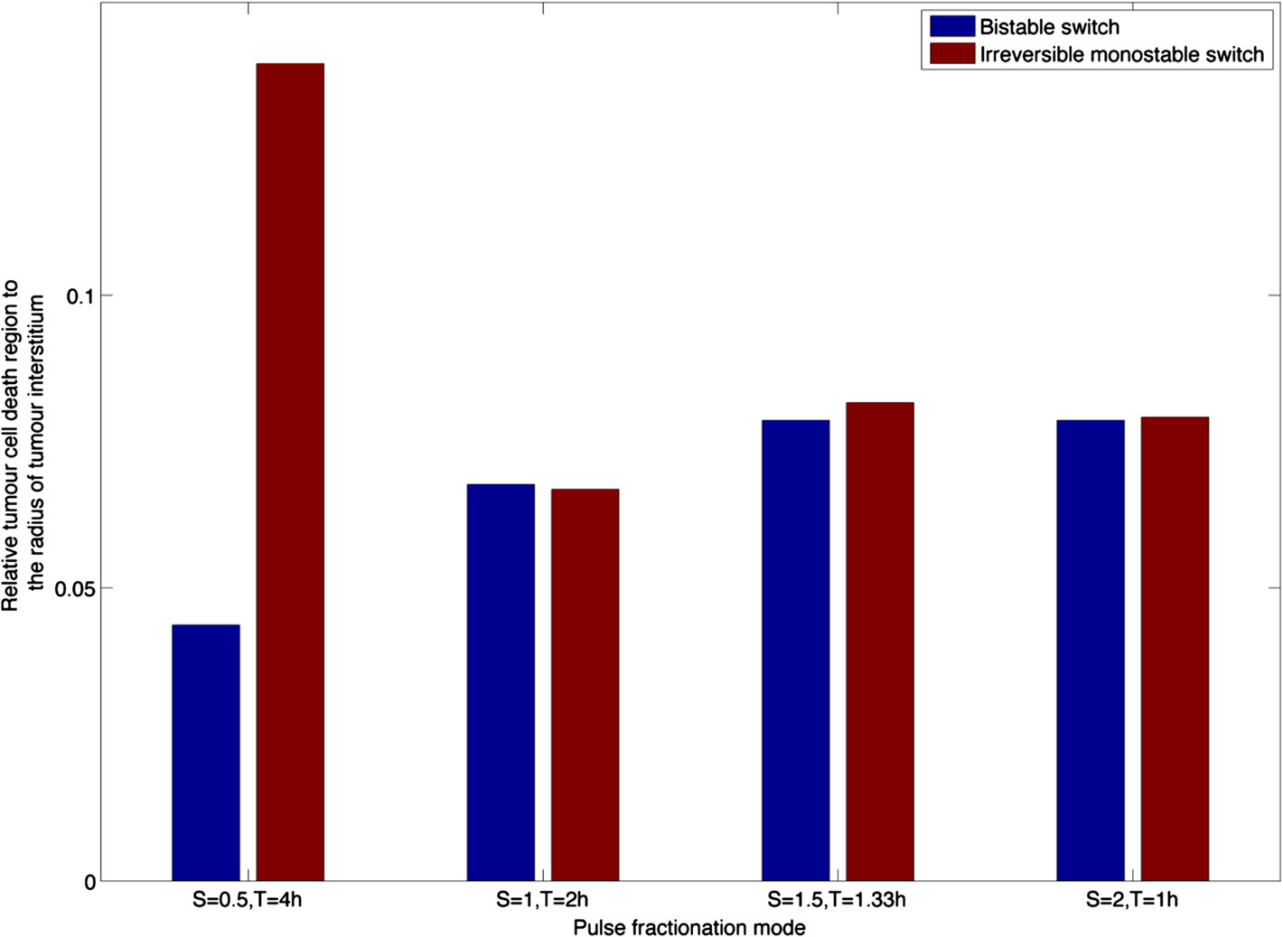
Effect of pulse fractionations on the relative tumour cell death region at 10 h.

Also examined is the width of tumour cell death region in response to different pulse fractionations for a fixed product of pulse strength and pulse duration. Figure 8 shows similar results for different pulse fractionations except for the case with *S* = 0.5, *T* = 4 h, where contrasting results are observed for the bistable and monostable switch. At this pulse fractionation, the monostable switch predicts a much wider cell death region than that by the bistable switch. The difference between bistable and irreversible monostable switches may be due to the following reasons: (i) different intracellular apoptosis signalling dynamics; (ii) different intracellular drug concentrations determined by interstitial drug transport and reactions or; (iii) a combination of both. These are examined further as detailed below.

**Figure 9:**
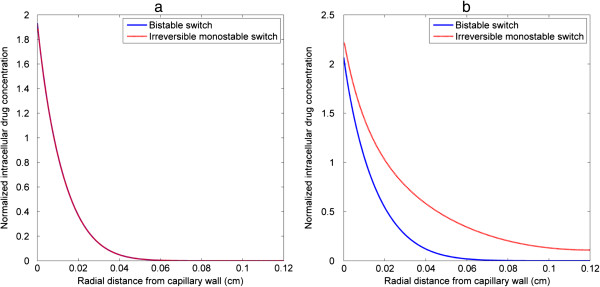
**Time evolution of intracellular drug concentration under one specific pulse fractionation.** Snapshots of cross-sectional profiles at z/L = 0.5 of intracellular drug concentration for bistable and irreversible monostable switches under pulse injection *S* = 0.5, *T* = 4 h at **(a)** t = 2.5 h, **(b)** t = 4 h.

**Figure 10:**
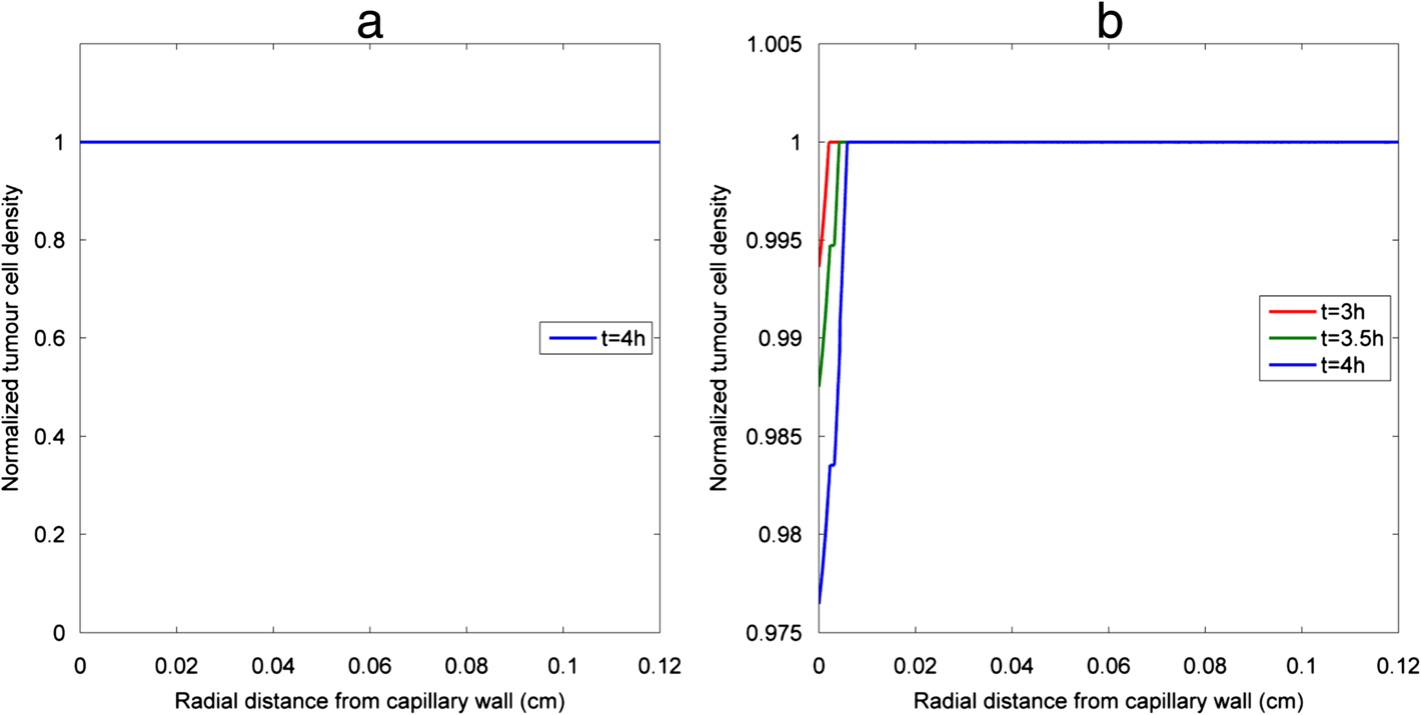
**Time evolution of tumour cell density under the same pulse fractionation.** Snapshots of cross-sectional profiles at z/L = 0.5 of tumour cell density under the same pulse fractionation as in Figure 9 for **(a)** bistable switch, **(b)** irreversible monostable switch.

Examined first are the intracellular drug concentrations which act as upstream stimuli to trigger cell apoptosis. Intracellular drug concentrations at two time points during the injection are shown in Figure 9. At an earlier time (t = 2.5 h in Figure 9(a)), intracellular drug concentration profiles are identical for both cases, but at the end of injection (t = 4 h in Figure 9(b)), intracellular drug concentrations with the monostable switch are apparently higher than those with the bistable switch, and the region where intracellular drug concentrations are above the threshold is wider. Temporal snapshots of tumour cell density are displayed for both cases in Figure 10. Obviously, there is no sign of cell death during the injection period for the bistable switch, but for the irreversible monostable switch cell density starts to decrease at 3 h. The falling tumour cell density triggers the feedback loop: decreased cell density leads to reduced drug consumption, which allows further drug transport and accumulation inside the cells, thus leading to cell death in the further region. The strong coupling between drug transport and tumour cell density is not only demonstrated by the numerical results shown here, but can also be shown by performing a non-dimensional analysis of the extracellular drug transport equation. In addition, Zheng *et al.*[27] have provided experimental evidence that drug-induced apoptosis facilitates drug penetration in solid tumours. Overall, we can conclude that there may exist small differences in response due to differences in intracellular dynamics (this was also encountered in [46]), and that the simulations reveal how transport and cellular effect may be coupled bidirectionally.

### Sensitivity analysis

Parameters used in the mathematical models are either related to drug transport or involved in drug effect. With regard to drug transport, parameters can be further divided into three groups: (i) diffusion related parameters, namely drug diffusivity (*D*), diffusive permeability (*P*), (ii) convection related parameters, such as hydraulic permeability (*L*_*p*_*),* hydraulic conductivity in the interstitium (*K*), (iii) tumour geometric parameters, for example, the radius of blood vessel (R_C_) and tumour interstitium (R_T_).

Based on the analysis presented above (in drug transport section), it is deduced that convection plays a minor role in the transmural and interstitial drug transport. Since convection is dependent on hydraulic conductivity *L*_*p*_ and tissue hydraulic conductivity *K*, their effects on blood flow are examined, and both are found to have a marginal effect on enhancing the transmural velocity (*J*_*F*_) (results not shown here).

Parameters involved in the intracellular signalling models are estimated to reflect a time scale and threshold value in a reasonable range, which agree qualitatively with those obtained from a cascade of signal transduction. Overall, transparent effects of these parameters are observed as expected from the coarse-grained intracellular signalling models. A slower kinetic rate or an elevated threshold would make it more difficult to trigger apoptosis while in the opposite scenario, relieving the constraints of apoptosis may exert a further effect on improving interstitial drug transport. As the study is oriented towards an integrative understanding of drug effect with account for mechanistic drug action, the intracellular signalling models together with the estimated parameter values adopted in the present study are sufficient to serve the purpose.

Therefore, the sensitivity study presented here is focused on how interstitial drug transport may be perturbed by altering diffusion related parameters, namely drug diffusivity and diffusive permeability, and geometric parameter (the size of tumour interstitium).

#### Effect of drug diffusivity

**Figure 11:**
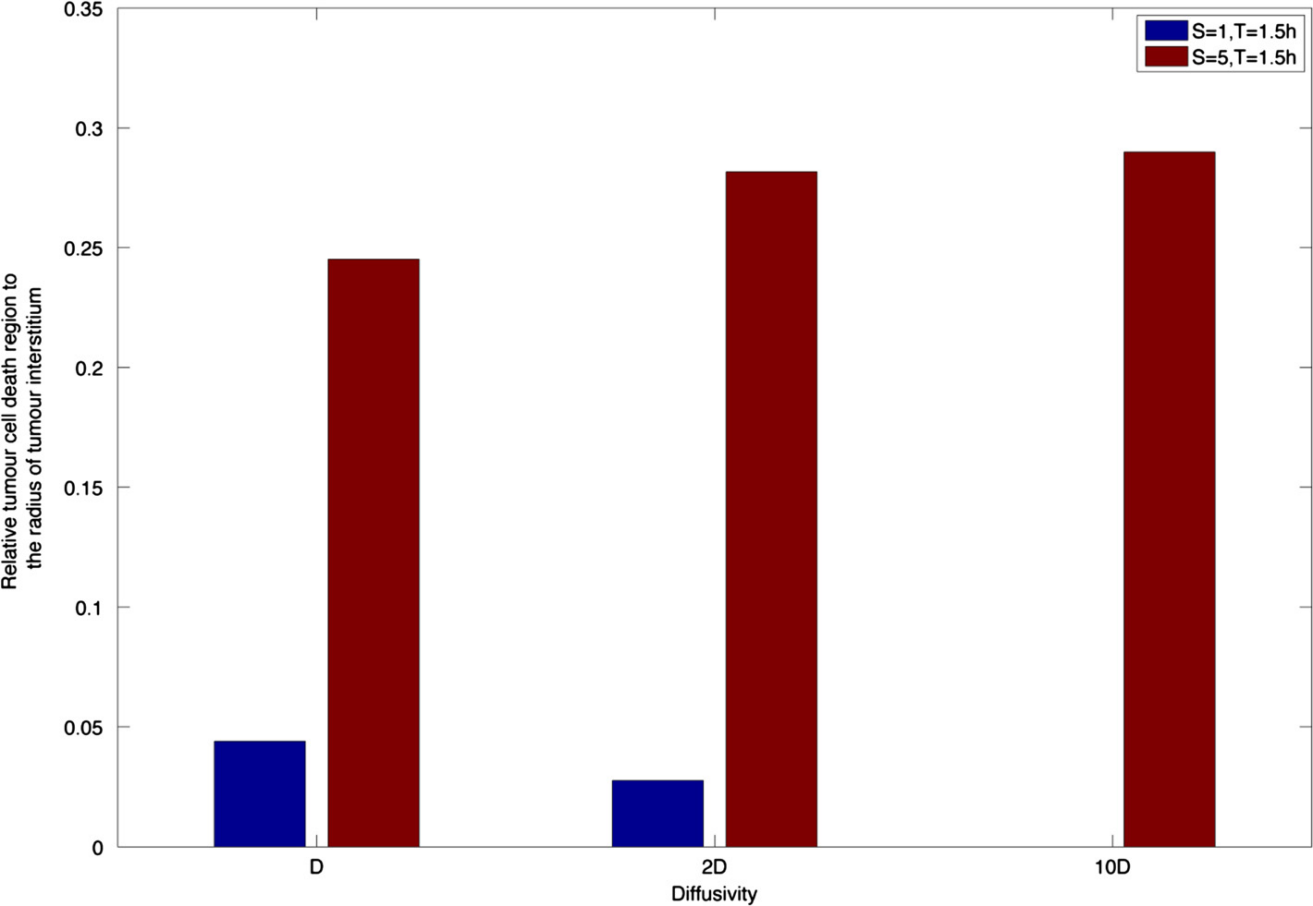
Effect of drug diffusivity on the relative tumour cell death region at 10 h for bistable switch.

The effect of increasing drug diffusivity (1*D*, 2*D* and 10*D,* where *D* is the value of diffusivity in the baseline case) is studied for two different pulse intensities. It is worth noting that the influence of drug diffusivity on the tumour cell density also depends on the intensity of pulse injection. As shown in Figure 11, increasing drug diffusivity can lead to reduced cell killing for pulse injection, with failure to trigger apoptosis at 10 *D* at normal intensity. Higher drug diffusivity allows the drug to transport further beyond the immediate vicinity of the vessel wall, and may help to establish a homogeneous concentration profile. However, it is not as simple when examined together with the specific requirement for apoptosis that the intracellular drug concentration needs to be sustained above its threshold for a sufficient length of time. For a smaller amount of drug, increased drug diffusivity somewhat dilutes the drug concentration, making it more difficult to satisfy the condition for apoptosis; as a consequence, the cell killing region is reduced. In this context, it would be favourable if the drug is concentrated in a limited region to exercise its effect locally. For a higher dosage, the opposite scenario occurs where the cell death domain is extended due to improved drug distribution as illustrated in Figure 11. An obvious increase in the width of cell death region is found when drug diffusivity is doubled, but further increase in diffusivity produces little change. The simulation results indicate that the effect of drug diffusivity needs to be examined by considering the balance between interstitial drug transport and the specific requirement of intracellular apoptosis dynamics, and it is dependent on the dosage applied. Similar trends are observed when the monostable apoptosis switch is employed. Overall, we see from the integrated study that the effect of drug diffusivity on the outcome is not necessarily as simple as may be expected by analysing diffusion in isolation.

#### Effect of diffusive permeability

**Figure 12:**
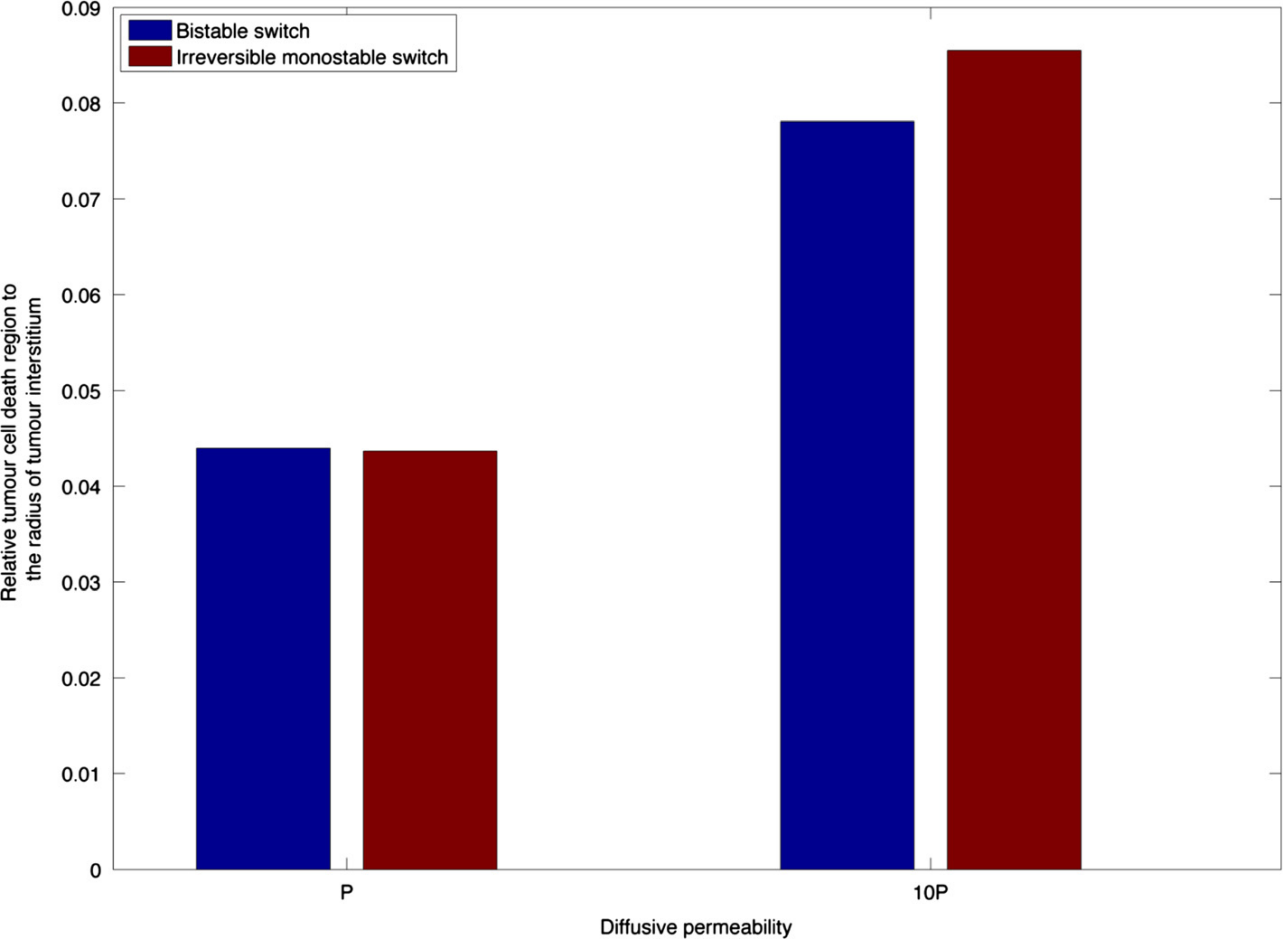
Effect of diffusive permeability on the relative tumour cell death region at 10 h.

Drugs extravasate across the blood vessel wall by diffusion and convection with the former being the dominant mode. The diffusive transmural flux is determined by the diffusive permeability of the drug and the concentration gradient across the vessel wall. Examined here is the distribution of the tumour cell density for the baseline diffusive permeability (*P*) and a much higher permeability (10*P*) under a given pulse. As shown in Figure 12, an increase in diffusive permeability results in an extension of the cell death region, but it is still limited to a narrow region close to the vessel wall even when the diffusive permeability is increased by ten-fold. This may be attributed to the following: (1) interstitial drug transport represents an obstacle in transporting excessive drugs away from the vessel wall; (2) more drugs are transported back to blood vessel due to the reversal concentration gradient caused by the termination of pulse injection.

#### Effect of the size of tumour interstitium

**Figure 13:**
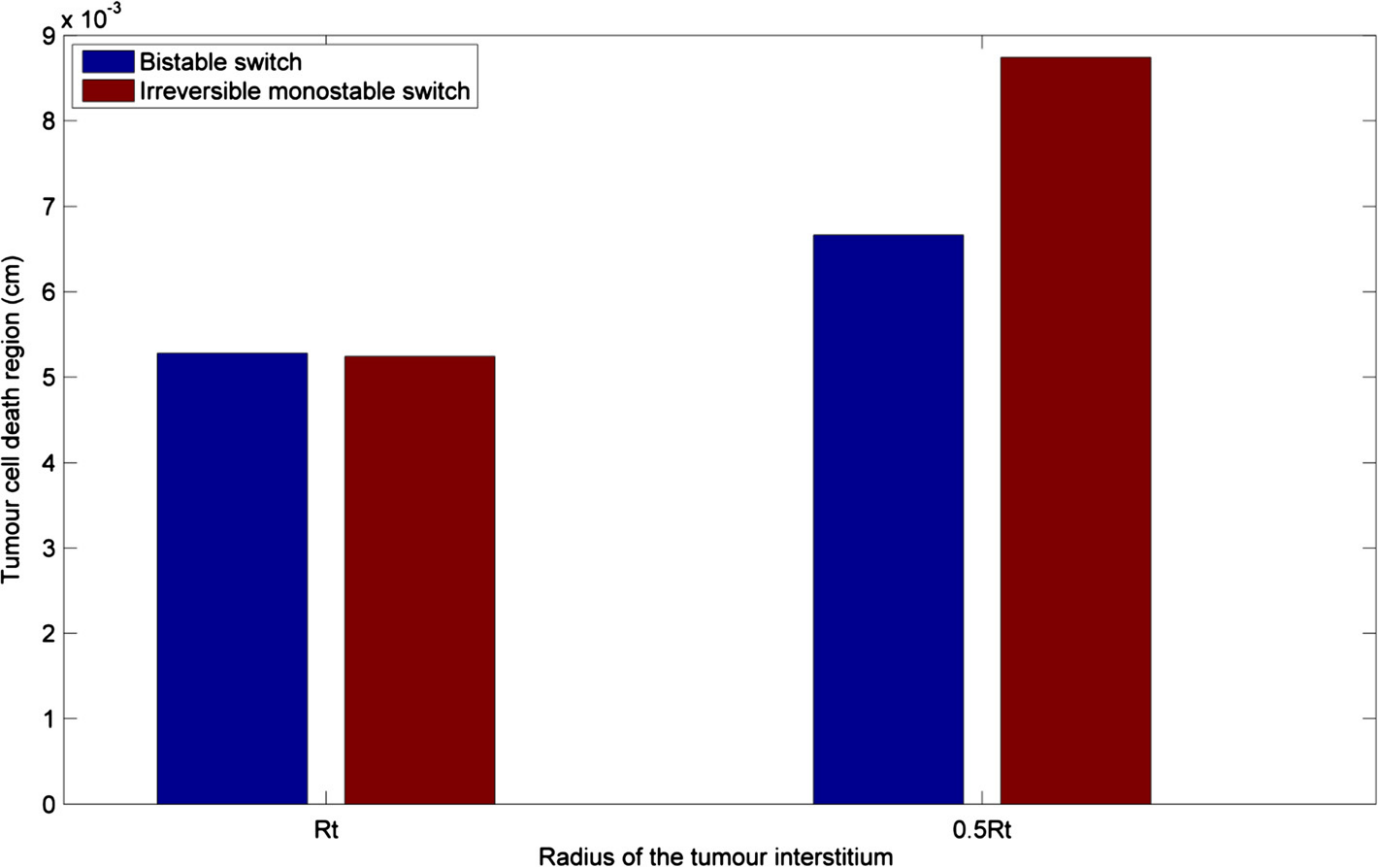
Effect of the size of tumour interstitium on the tumour cell death region at 10 h.

When the size of the tumour interstitium is reduced by half, the region of tumour cell death is still confined to the proximal region to the vessel wall with a marginal increase as shown in Figure 13. This is explained as follows. Overall, the reduced size (over a broad spectrum of tumour size) has negligible effect on interstitial drug transport during the injection phase. However, during the post-injection phase, the effect of a reduced tumour size can be seen in terms of the enhanced convective transmural flux (as a result of increased transmural velocity), which partially compensates for the negative diffusive flux back to the blood vessel, and allows more drugs to be retained in the vicinity, thus leading to enhanced penetration in the interstitium. With even smaller tumour sizes, the effect of transport limitation is gradually removed. Analysis of varying tumour sizes demonstrates that the effect of drugs is determined by the interaction between multiple drug transport processes and intracellular signalling dynamics.

## Discussion

In this study, the first steps have been taken towards developing an *in silico* experimental platform with integration of blood flow, drug transport (both vascular and interstitial) and cellular signalling dynamics to provide an overall framework to systematically evaluate the effect of anticancer drugs on tumour cells. This platform, described by our model, contains the minimal essential elements to understand the basic aspects of the interplay between drug transport and cellular effects. The model set-up represents an idealized tumour with a well controlled environment, which is essential for understanding the interactions of many complicating factors. The tumour cell density (the output) is determined by the combined action of drug input (determined by tumour blood flow and drug transport) and intracellular signalling (involving non-linear dynamics), when subject to a particular form of drug stimulus (the input), which is itself determined by the mode of delivery (not modelled in detail). Basic descriptions of the processes involved are incorporated with an aim to create a model system which captures the information flow from drug delivery to causal effect, and is capable of serving as a platform to understand the interplay between transport and extracellular factors on one hand and cellular features on the other hand.

### Drug transport

Rather than treating the tumour blood as a homogeneous source term in the transport equation, the models describe tumour blood flow explicitly by coupling vascular blood flow with the interstitial fluid flow through an elevated tumour vascular hydraulic conductivity. Drug delivery is subsequently examined following the same transport routes. From the simulation results, few differences can be observed between the drug concentration profiles at different axial locations, indicating that intravascular transport (convection dominant) is not a rate limiting step in the drug transport processes. Of course it must be borne in mind that this finding is based on the highly simplified geometry adopted in this study – a single vessel similar to the Krogh model. It is acknowledged that a single vessel model is not always the best model; it fails to capture the heterogeneous distribution of functional blood vessels in tumours that result in large avascular regions. However, the present study focuses on providing essential and clear-cut insights into drug distribution and drug effect by conceptualizing the information flow in an integrated manner. Potentially important factors, such as the complex tumour vascular geometry (or even non-Newtonian blood rheological properties) may significantly increase resistance to tumour blood flow, thus leading to insufficient drug delivery through the tumour vasculature and even more heterogeneous drug distribution in the tumour interstitium. A description of more realistic tumour vascular network, based on advanced imaging techniques, could potentially be incorporated into the modelling framework for more realistic prediction of drug concentration distribution in specific tumours. We also note that to bring new insights into drug delivery through a complex vascular network in the tumour, it is necessary to first understand what the effect is in a simpler vasculature.

The higher vascular permeability normally observed in tumour tissues facilitates transmural transport of drugs into the tumour interstitium. However, it is noted that drug penetration is restricted to the region close to the blood vessel, with poor drug distribution in the interstitium. With regard to drug transport into tumour cells, in the case of the specific anticancer drug selected (doxorubicin), it is found that the drug is preferentially sequestered in tumour cells. Therefore, limited drug penetration in the interstitium is a major difficulty to overcome in order to improve drug efficacy. Compared to drug diffusion in the interstitium, drug consumption by tumour cells determined by the tumour cell density plays a dominant role in impairing interstitial penetration (discussed later). On the other hand, the heterogeneous drug distribution found in the spatially distributed system also implies that homogeneous compartment models may not be sufficient for accurate predictions of drug efficacy. It is worth pointing out that the modelling framework developed is not only applicable to chemotherapeutic agents, but (with minor modifications) also to oxygen and other endocrine signals, which share the same transport pathways. Further, interstitial drug transport may be implicitly affected by cellular responses, such as the presence of low pH due to the adaptive metabolism towards oxygen starvation, and detoxifying anticancer drugs due to acquirement of drug resistances. Understanding the interplay of such factors is beyond the scope of this study, but can be built on the existing framework presented here.

### Drug effect

Drug response is evaluated broadly by empirical or mechanistic approaches. This study is motivated by the desire to provide a mechanistic understanding of drug effect by taking into account the interactions between drugs and targets, the downstream signalling reactions and ultimately cell fate decision making. In the current model, apoptosis is the primary cellular response triggered by anticancer drugs and dynamic analysis of drug effect is performed at both cellular and tissue level.

It is important to choose an appropriate level of cellular effects to be included in the model given the fact that many of the biochemical details are still unknown (or may be questionable or may vary between cell types). Coarse grained descriptions of intracellular process are adopted here, with a view to qualitatively capture the nature of signal transduction in the cell and to retain the relevant input-output signalling characteristics. Future work to include more biochemical details of the signalling network (including the detailed dynamics of the caspase network and its regulation) is needed in order to obtain a more detailed depiction of the cellular signalling and understand the roles of multiple intracellular regulatory mechanisms.

In the current work, two types of apoptosis modules with qualitatively distinct dynamics of signal transduction are included and examined separately: a bistable switch and an irreversible monostable switch. The two models are examined to address whether such different dynamic characteristics at the cellular level would result in different drug effects at the population level. It has been found that in most respects the two models predict broadly similar effects. A related point to be made is that when cellular signalling is included, one should be very cautious about claims of validating models from scanty data. The extent to which signalling models can be distinguished in such a context is discussed elsewhere [46]. It is worth emphasizing that our intracellular models are essentially minimal models but have the features which would be expected of all apoptosis models.

### Drug transport vs. drug effect

Our analysis reveals that in pulse injections, the drug killing effect is typically confined to the proximal region due to limited drug penetration for both types of apoptosis models (Figures 5,6,7). Our analysis reveals in multiple ways, the need for incorporating the dynamics of intracellular signalling in mechanistic predictions of drug effect and investigating drug transport and drug effect in an integrated manner. Within the present modelling framework, it is found that interstitial drug transport and drug effect are strongly coupled: drug-induced pharmacological effect (apoptosis) can enhance drug penetration in the interstitium, which constrains the exertion of drug action. This indicates that an optimal staged treatment schedule might help to result in a rapid penetration and the subsequent cell killing in regions further away from blood vessels.

It is natural to expect that greater diffusivity enhances drug penetration in the interstitium, resulting in a more homogeneous distribution of drug concentration. However, as our analysis shows, it is not necessarily the case that greater diffusivity would improve drug effect on tumour cells for a given stimulus. This is due to the specific requirement for apoptosis being triggered and the interplay between intracellular dynamics and interstitial drug transport. Drug diffusivity in tumour tissues is not only related to the physiological properties of anticancer agents, i.e. their charge, size or shape, but also to the structures of tumour tissues [50]. Therefore, the analysis presented here may help to refine drug infusion strategies by noting the different effects of drug diffusivity in a drug- and tumour- specific context.

## Conclusions

In this paper, an *in silico* experimental platform is employed, which describes the flow of information from drug delivery to drug effect combining tumour blood flow, anticancer drug transport and cellular dynamics. Within the simplified model setting, a series of investigations on different drug stimuli and parameters is presented, providing explicit insights into the effect of drug and the interplay between multiple transport processes and intracellular signalling dynamics. Although the modelling platform in the current study is coarse-grained and qualitative in nature, it is capable of accommodating other mathematical models and allowing for fine graining and augmented descriptions of individual subprocesses systematically. Quantitative understanding of the contributing factors could be achieved for specific tumour types and specific anticancer drugs, in conjunction with the relevant experimental data at multiple scales. The modelling platform can serve as a computational tool to perform a thorough sensitivity analysis for the control and optimization of chemotherapeutic processes.

## Competing interests

The authors declare that they have no competing interests.

## Authors’ contributions

CL, JK and XYX planned the paper, CL performed the computational work, CL wrote the paper with input by XYX and JK. All authors read and approved the final manuscript.
